# Explant Modeling of the Immune Environment of Head and Neck Cancer

**DOI:** 10.3389/fonc.2021.611365

**Published:** 2021-06-17

**Authors:** Shay Sharon, Thomas Duhen, Shelly Bambina, Jason Baird, Rom Leidner, Bryan Bell, Nardy Casap, Marka Crittenden, Swetha Vasudevan, Maria Jubran, Nataly Kravchenko-Balasha, Michael Gough

**Affiliations:** ^1^ Department of Oral and Maxillofacial Surgery, Hadassah and Hebrew University Medical Center, Jerusalem, Israel; ^2^ Earle A. Chiles Research Institute, Robert W. Franz Cancer Center, Providence Portland Medical Center, Portland, OR, United States; ^3^ The Oregon Clinic, Portland, OR, United States; ^4^ The Institute of Biomedical and Oral Research, Hebrew University of Jerusalem, Jerusalem, Israel

**Keywords:** tumor, head and neck cancer, immunotherapy, explant, cytokine, PD1, OX40, CTLA4

## Abstract

Patients exhibit distinct responses to immunotherapies that are thought to be linked to their tumor immune environment. However, wide variations in outcomes are also observed in patients with matched baseline tumor environments, indicating that the biological response to treatment is not currently predictable using a snapshot analysis. To investigate the relationship between the immune environment of tumors and the biological response to immunotherapies, we characterized four murine head and neck squamous cell carcinoma (HNSCC) models on two genetic backgrounds. Using tumor explants from those models, we identified correlations between the composition of infiltrating immune cells and baseline cytokine profiles prior to treatment. Following treatment with PD-1 blockade, CTLA-4 blockade, or OX40 stimulation, we observed inter-individual variability in the response to therapy between genetically identical animals bearing the same tumor. These distinct biological responses to treatment were not linked to the initial tumor immune environment, meaning that outcome would not be predictable from a baseline analysis of the tumor infiltrates. We similarly performed the explant assay on patient HNSCC tumors and found significant variability between the baseline environment of the tumors and their response to therapy. We propose that tumor explants provide a rapid biological assay to assess response to candidate immunotherapies that may allow matching therapies to individual patient tumors. Further development of explant approaches may allow screening and monitoring of treatment responses in HNSCC.

## Introduction

Incorporation of immunotherapy into the treatment of head and neck squamous cell carcinoma (HNSCC) has brought major advancements in patient care (1). The question remains as to why a particular group of patients will benefit from immunotherapy, while others will progress. Biomarkers, such as programmed death receptor-ligand 1 (PD-L1), tumor mutational burden, and gene signatures demonstrating an inflamed tumor microenvironment are being evaluated to predict response to immunotherapy; however, it is clear that some patients who express a positive predictive biomarker will not respond to treatment and some who lack that biomarker will respond. Thus, a practical method of testing the effectiveness of therapeutic combinations on an individual tumor *ex-vivo* would be transformational to the field.

Preclinical tumor models allow for extensive manipulations of cancer cells and the host immune system, but the relatively homogenous genomic background of standard murine strains and the finite number of syngeneic cancer cell lines highlight the limitations in modeling the diversity of cancer-immune interactions seen in patients. It is also infeasible to test all potential immunotherapies and combinations in an individual patient. To understand how patient tumors control and are in turn controlled by the immune system, we need to develop a model that reflects cellular interactions observed in patients. This experimental system will require to take into account the heterogenic nature of the interrelationship between the tumor and its host, while also providing data to direct treatment.

Monitoring the proportions of immune-cells and concentrations of other circulating components in peripheral blood may provide some potential biomarkers (2, 3). However, there is only limited data demonstrating that changes in immune cell numbers in peripheral blood are also reflected in the tumor. In one recent study we found that while patients varied in their baseline level of myeloid and T cell populations in the peripheral blood, there was no correlation between the number of these cells in the blood and the number of their corresponding population in the tumor (4). These data suggest that while blood analysis can have value in patients (3), it cannot necessarily predict the tumor immune environment.

Several reports using preclinical models have shown that successful responses to checkpoint inhibitor-based immunotherapies were observed even when immune cell recirculation was blocked, indicating that circulating immune cells are not always necessary to induce tumor regression (5, 6). This suggest that at least some of the critical immune cells are already resident in tumors (7, 8) and tumor-specific T cell clones present in the tumor are detected at very low frequency in draining lymph nodes and the peripheral blood (7). In addition to the number and type of immune cells located in the tumor, it has been shown in HNSCC that the spatial relationship between suppressive and effector elements within a tumor can impact patient outcomes (9). Similarly, while the degree of T cell infiltration in the tumor has consistently been associated with good prognosis (10), a difference in outcome can be observed depending on whether the T cells were located amongst cancer cells, rather than in the tumor stroma (11). In HNSCC, when T cell infiltrate analysis was limited to those associated with cancer cells, T cell infiltrate was no longer prognostic (12). These data suggest that an intact tumor environment may react differently to therapeutic agents when compared to tumor digests, since specific cell-cell relationships are lost. If the tumor represents the critical environment where immunotherapies can exert their effect, then therapies should be studied when the tumor environment is intact. To address this, we previously developed a tumor explant model where tumor fragments are evaluated *ex-vivo* and used it to explore the variation in responses to tumor-restricted immunotherapies using STING ligands (13).

For these reasons, we evaluated explants of a panel of murine HNSCC models, characterizing features of their immune environment to identify linked elements that may impact their baseline immunobiology. In addition, we evaluated their *ex-vivo* response to treatment with distinct T cell-targeted immunotherapies. To determine whether this could be applied to tumor explants from patients with HNSCC, we performed a preliminary analysis in patients with very distinct immune environments. We propose that explant analysis can provide rapid information as to the biological response of patients to candidate immunotherapies, and could guide optimal, personalized immunotherapy interventions for patients with HNSCC.

## Materials and Methods

### Ethics

All animal protocols were approved by the Earle A. Chiles Research Institute IACUC (Animal Welfare Assurance No. A3913-01). De-identified human tissue was obtained under IRB# 12-075 approved by the Providence Portland Medical Center IRB.

### Animals and Cell Lines

6 to 8-week-old female C57BL/6 mice (stock# 000664) and C3H mice (stock# 000659) were obtained from The Jackson Laboratory, and experiments were performed on 3 to 5 mice per group. The SCC-VII squamous cell carcinoma cell-line was kindly provided in 2014 by Dr. Lee (Duke University Medical Center, Durham, NC). The TC1 squamous cell carcinoma cell-line was kindly provided by Dr. Hong Ming Hu (EACRI, Portland, OR). The Moc1 and Moc2 murine HNSCC cell-lines (14) were kindly provided in 2017 by Dr. Ravindra Uppaluri (Dana Faber Cancer Institute, MA). Moc1 cells were transfected with a GFP-SIINFEKL fusion protein to ensure non-secreted cytoplasmic expression of the model antigen, or GFP alone, to generate Moc1-ova or Moc1-GFP and sorted for cells with stable expression of GFP. The presentation of SIINFEKL was confirmed using a B3Z T cell assay (15). Species identity checks on these murine cell-lines were performed with murine-specific MHC antibodies and were tested for contamination within the past 6 months using a Mycoplasma Detection Kit (SouthernBiotech, Birmingham, Alabama). Moc1, Moc1-ova, Moc2, and TC1 tumors were established in immune-competent C57BL/6 mice, and SCC-VII tumors were established in C3H mice at a dose of 1x10^6^ Moc1 cells, 1x10^6^ Moc2 cells, 5x10^5^ SCC-VII cells, and 1x10^5^ TC1 cells. Inoculation of the highly immunogenic Moc1-ova cells was subsequently followed by 3 doses of anti-CD40L at days 0,1, and 2 to eliminate the immune response at tumor challenge and permit tumor formation (6). Tumors were allowed to develop to 12 mm in diameter, at which point the tumors were harvested and analyzed in parallel by explant assay, multiplex IHC, and flow cytometry.

### Antibodies and Reagents

Flow cytometry antibodies for murine samples included CD3e-PE, PD1-BV605, CD45-BV786 (BDbiosciences), CD90.1-FITC, CD62L-PECy7, CD45.1-PE (ebioscience), CD45.2-Alexa700, CD-8α-Percp Cy5.5, CD4-FITC, CD103-APC, CD24-APCcy7 (Invitrogen), CD44-APCy7, CD4-BV421, F4/80-PercpCy5.5, CD39-PECy7, PDL1-PE, CD90.2-Alexa 700, MHCII-BV421, CD11b-BV650, Ly6C-BV711 (Biolegend), CD8α-PE-Texas red (life technologies), CD278 (ICOS)-PE (Biolegend). Flow cytometry antibodies for human samples included CD45-BV510, CD3-Alexa700, CD4-BV785, CD8-BV711, CTLA-4-PE/Dazzle 594, 4-1BB-PE (Biolegend), CD39-BV650, PD-1-PE-Cy7 and Ki-67 Alexa 488 (BD Biosciences), CD103-APC and FOXP3-Alexa 700 (eBioscience).

Mouse antibodies for the explant assays included anti-PD1 (RMP1-14) and anti-CTLA-4 (9D9) by BioXCell and OX40 (OX86) kindly provided by Dr. Andrew Weinberg (EACRI). Human antibodies for the explant assays included anti-PD1 and anti-CTLA4 purchased from Invitrogen and anti-OX40 kindly provided by Dr. Andrew Weinberg (EACRI).

### Preparation of Explants

Fresh tumor samples were delivered on ice to the laboratory, and processing of the tissue into explants began immediately. We developed two methods of producing explants: 1. Dissection of the tumor using a #10 blade into cuboidal 1.5-2.0 mm fragments; 2. A circular punch-biopsy of the tumor is placed onto a carrier that is submerged in ice-cold PBS followed by vibrating blade microtome (Leica VT1200) dissection into 300µm thick slices. The prepared fragments are kept in ice-cold PBS until completion of sectioning, followed by placement of each fragment into 96-well plates with media alone or media containing an immunotherapeutic agent. After 24 hours of incubation at 37°C, the supernatant media is collected and analyzed for cytokine secretion by multiplex bead assay.

To include both intra-tumoral variability and variability between individual animals, each tumor type was sampled from 3 animals, each dissected into 16 explants, giving rise to a total of 48 explants that were divided into 4 groups of 12 replicates.

### Multiplex IHC

Tumors grown in C57BL/6 and C3H mice were harvested upon reaching 12 mm in diameter and were fixed in zinc-based fixative for 24 hours at room temperature as previously described (16). Tissue was then processed by preparing 4 µm thick tissue sections, incubation of slides at 37°C and deparaffinization. The protocol included blocking with goat serum (Vector) prior to staining and dilution of primary antibodies in Renaissance Background reducing Diluent (Biocare medical). Tissue sections were boiled in Rodent Decloaker (Biocare Medical) for antigen retrieval, except prior to CD8 staining where pH=9 buffer (Perkin Elmer) was used. Primary antibodies were anti-CD3 (SP7, Abcam, Cambridge, UK), anti-CD8α (4SM15, eBioscience), anti-FoxP3 (FJK-16s, Invitrogen, Carlsbad, CA), anti-PD-L1 (D5V3B, Cell Signaling Technology, Danvers, MA), and DAPI (Perkin Elmer). Opal 7-Color Automation IHC Kit was used (690 for CD3, 570 for PD-L1, 620 for CD8α, and 520 for FoxP3). Slides were scanned with Vectra Polaris (Perkin Elmer, Waltham, MA) and analyzed using QuPath 0.2.0-m4 (17).

### Flow-Cytometry

Murine tumors grown in C57BL/6 and C3H mice were harvested upon reaching 12 mm in diameter, and single-cell suspensions were prepared by dissection into fragments manually and using a gentleMACS dissociator (Miltenyi Biotech, Bergisch Gladbach, Germany), followed by agitation in digest solution (250U/mL collagenase, 30U/mL DNase, 5mM CaCl_2_, 5% FBS in HBSS in PBS) for 30 minutes at 37°C. The digest was filtered through 100 μm nylon mesh to remove macroscopic debris. Cells were surface stained with the phenotypic markers and CD45, CD90.2, CD39, CD103, CD4, CD8, CD11b, MHCII, Ly6C, CD11b, CD24, PD1, PD-L1, and F4/80 to distinguish live CD11b^+^MHCII^+^Ly6C^-^F4/80^+^ macrophages, CD11b^+^MHCII^-^Ly6C^+^ neutrophils, CD11b^+^F4/80^-^Ly6C^+^ monocytic cells, and CD11b^+^MHCII^+^Ly6C^-^CD24^+^ dendritic cells as previously described (13, 18). For analysis of explants following treatment, individual treated tissue fragments were dissociated as above and surface stained with the phenotypic markers CD45, CD90.2, CD4, CD8, CD25, and ICOS.

Human tumor specimens were prepared as follows: under sterile conditions, tumors were cut into small pieces and digested in RPMI-1640 supplemented with hyaluronidase at 0.5 mg/mL, collagenase at 1mg/mL (both Sigma-Aldrich), DNase at 30 U/mL (Roche) as well as human serum albumin (MP Biomedicals) at 1.5% final concentration. Cells were digested for 1 hour at room temperature under agitation with a magnetic stir bar. Cells suspensions were filtered through a 70 µm filter. Tumor single-cell suspensions were cryopreserved until further analysis. Samples were run on a BD LSRFortessa flow cytometer or BD LSRII flow cytometer (BD Biosciences) and analyzed using FlowJo (Tree Star, Ashland, OR).

### Statistical Analysis

Data were analyzed and graphed using Prism (GraphPad Software, La Jolla, CA). Individual data sets were compared using the two-tailed unpaired Student t-test. Initial clustering of samples based on infiltrating cell types was performed using ClusterVis (19). Principal components are calculated as described (20) using ClusterVis (19). Missing data is assigned using Singular Value Decomposition with imputation iteratively until estimates of missing values converge. Correlations between immune cells and cytokine and chemokine production were determined as Pearson correlation coefficients.

### Surprisal Analysis

The expression level of a molecule (in our case, a cytokine) is decomposed by the surprisal analysis into its expected expression level at the steady state, and into its deviation from it due to environmental or genomic constraints (21, 22). Any constraint that alters a part of the secreted protein network structure in the system, which in turn, causes specific group of secreted proteins (=subnetwork) to undergo coordinated changes in their expression levels is defined as an unbalanced process. Each of the altered secreted proteins can be involved in several unbalanced processes due to the non-linearity of biological networks. To decompose protein expression levels into the levels at the steady state and deviation thereof, the following equation is utilized: $\ln X_{i}(k)=\ln X_{i}^{o}(k)-\sum_{\alpha =1}G_{i\alpha }\lambda_{\alpha }(k)$ (21, 22). *X_i_*(*k*) is the actual, experimentally measured expression level of the protein i in a cancer sample k. $X_{i}^{0}$ is the expression levels at the steady state. In cases where $X_{i}(k)\neq X_{i}^{0}$, we assume that the expression level of protein i was altered due to constraints that operate on the system. The analysis uncovers the complete set of constraints in each tumor, including the secreted proteins that are affected by these constraints and have therefore deviated from their steady state levels. Each constraint significantly influences only a subset of proteins (unbalanced process) in a similar way by causing the collective deviations of the protein levels (upward or downward) from the balanced level (23).

Several unbalanced processes may operate in each sample and their number is determined as described previously (22, 24), indexed by α = 1,2,3…. The term $\sum_{\alpha =1}G_{i\alpha }\lambda_{\alpha }(k)$ represents the sum of deviations in expression level of protein i due to the various constraints, or unbalanced processes that exist in the sample. The *λ_α_*(*k*) values, denoting the amplitude of each unbalanced process, in every sample *k*, i.e. the extent of the participation of each unbalanced process α, in every sample *k*. The amplitude, *λ_α_*(*k*) values, denoting the extent of the participation of each individual protein *i* in the specific unbalanced process, *α*.

The functional subnetworks were generated using a python script as previously described (23). The goal was to generate a functional network according to STRING database, where proteins with negative G values are marked red and proteins with positive G values are marked blue, to easily identify the correlations and anti-correlations between the proteins in the network. The term *G_iα_*denotes the degree of participation of the protein *i* in the unbalanced process α, and its sign indicates the correlation or anti-correlation between proteins in the same process. For example, in a certain process α, proteins can be assigned the values: G_protein1,α_ = -0.50, G_protein2,α_ = 0.24, and G_protein3,α_ = 0.02, indicating that this process altered proteins 1 and 2 in opposite directions (i.e. protein 1 is upregulated and protein 2 is downregulated, or vice versa due to the process α), while not affecting protein 3. Note that each protein can take part in a number of unbalanced processes at once.

Importantly, not all processes are active in all tumors. The term *λ_α_(k)* represents the importance of the unbalanced process *α* in the tumor *k*. Its sign indicates the correlation or anti-correlation between the same processes in different tumors. For example, if the process α is assigned the values: *λ_α_(1)* = 3.1, *λ_α_(2)* = 0.02, and *λ_α_(5)* = 2.5, it means that this process influences the tumors of the patients indexed 1 and 5 in the same direction, while it is inactive in patient 2. To calculate an induction or reduction due to the process *α*, a product *G_iα_λ_α_(k)* is computed.

## Results

### Characterization of the Tumor Environment in Murine HNSCC

We characterized the tumor immune environment of murine HNSCC using four tumor models on two genetic backgrounds. Moc1, Moc2, and TC1 tumors were established in immune-competent C57BL/6 mice, and SCC-VII tumors were established in immune-competent C3H mice. In addition, Moc1 tumors engineered to express the model tumor antigen SIINFEKL (Moc1-ova) were included to evaluate the consequence of strong antigenicity. To directly link each measure to an individual tumor, all fresh tumors were initially bisected at a plane that divided each tumor into 40% and 60% counterparts. The smaller part was transferred into zinc-based fixative for IHC, while the larger part was bisected again into 40%/20% fragments for flow cytometry and explant analysis, respectively. The explant portion was sliced into 1.5-2.0mm cuboidal fragments that were individually seeded to wells of a 96-well plate, producing 16 replicates in total from each tumor.

**Figure 1:**
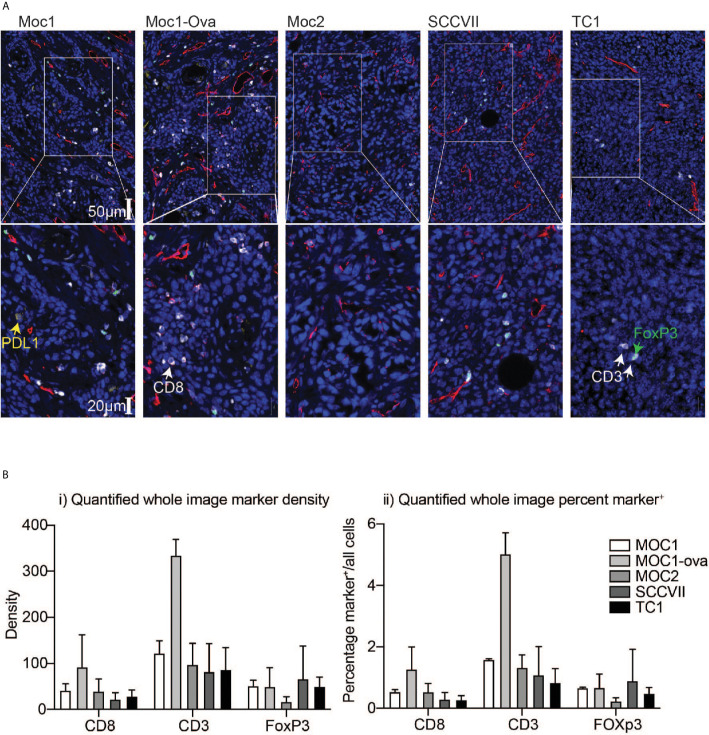
Differential immune infiltration across murine HNSCC tumor models by immunohistology. Tumors were established in immune-competent animals and tumors were harvested for analysis of infiltrating immune cells by IHC. **(A)** Representative images following multiplex IHC for infiltrating immune cells in the tumor showing PDL1 (yellow), CD3 (white), CD8 (red), FoxP3 (green), DAPI, (blue). Lower images are higher magnification inset of upper images. Scale bars are shown. **(B)** Quantification of i) marker density and ii) percent marker positive across tumor models. Graphs show means and SD of two tumors per group.

To understand the relationship between infiltrating immune cells in the tumor, the fixed tumors were paraffin-embedded and analyzed in parallel by multiplex IHC. Moc1-ova exhibited a prominent adaptive immune infiltrate, which was also evident to a lesser degree in Moc1 (**Figure 1A**). The immune infiltrate was characterized by high amounts of CD3^+^, CD8^+^, and Foxp3^+^ cells. A similar infiltrate of Foxp3^+^ cells was also evident in SCC-VII and TC1 tumors, but CD8^+^ cells were less common in these tumors (**Figure 1A**). The immune infiltrate was poor in Moc2 tumors (**Figure 1A**), as previously described (13, 25), though several foci of highly concentrated CD3^+^ cells were evident at the tumor periphery (not shown). Whole-slide quantitative analysis corroborated these data, showing a high amount of CD3^+^ T cells in Moc1-ova, and a tendency towards more CD8^+^ T cells compared to other models, although this did not reach statistical significance (**Figure 1B**). The models did not significantly vary in Foxp3^+^ cell infiltrate.

**Figure 2:**
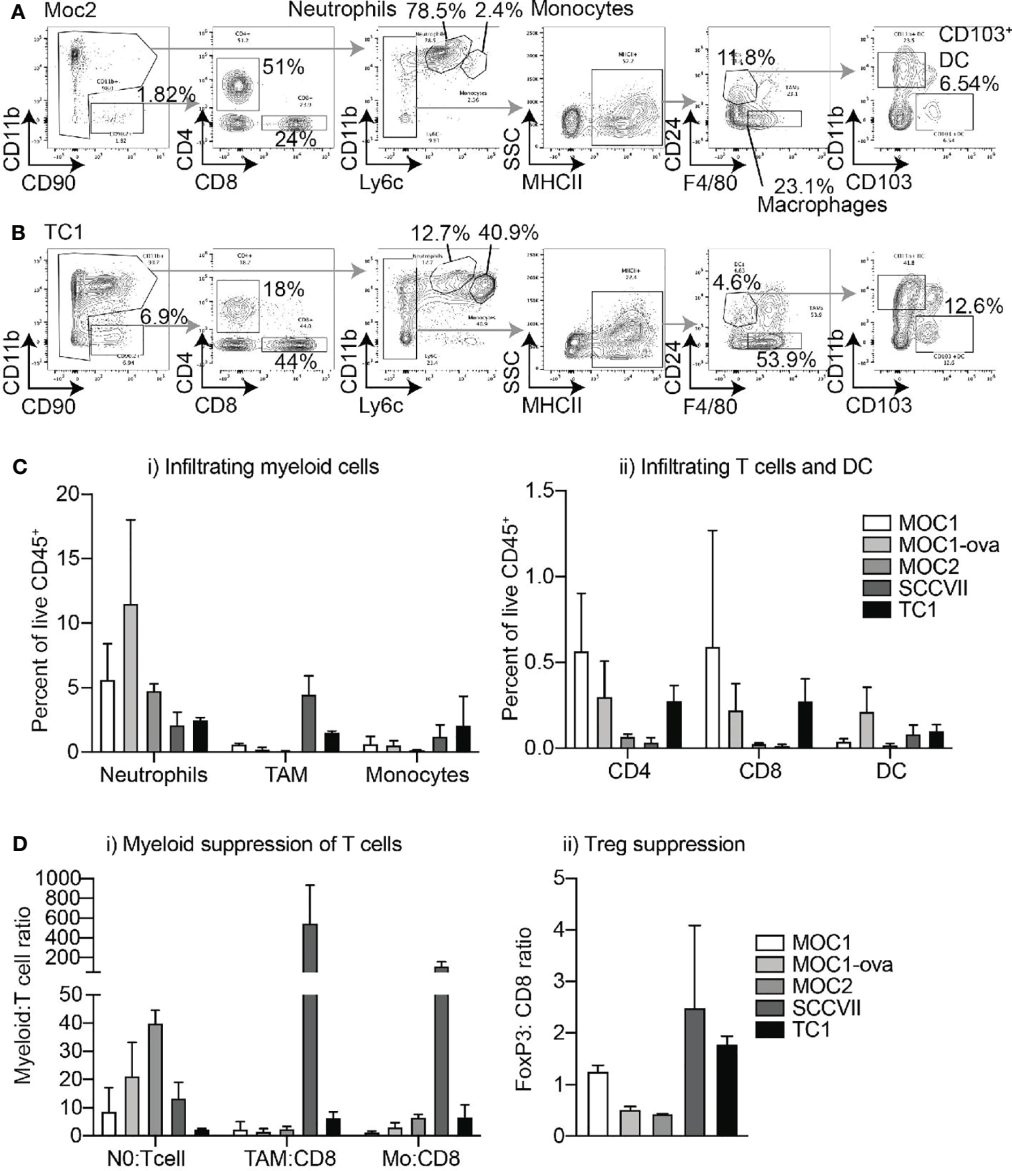
Differential immune infiltration across murine HNSCC tumor models by flow cytometry. Tumors were established in immune-competent animals and tumors were harvested for analysis of infiltrating immune cells by flow cytometry. Representative flow cytometry of **(A)** Moc2 and **(B)** TC1 showing gating strategies to identify individual cell types in two contrasting tumors. Subgating is shown using gray arrows, key populations are identified. **(C)** Quantified flow cytometry across multiple tumors for key populations of i) myeloid cells and ii) T cells and DC. **(D)** Relative levels of immune cells from **Figures 1** and **2** to show relative levels of i) myeloid cells and ii) Tregs to T cells to identify potential suppression patterns. 3-4 individual tumors per tumor type were analyzed.

The fresh matching specimens were digested to make single-cell suspensions and analyzed by multiparameter flow cytometry; gated on myeloid populations including F4/80^+^CD24^-^MHCII^+^Ly6C^-^ tumor-associated macrophages (TAM’s), CD11b^hi^Ly6C^int^Ly6G^hi^ neutrophils, CD11b^hi^Ly6C^hi^Ly6G^-^ monocytes, F4/80^-^CD24^+^MHCII^+^Ly6C^-^CD11b^-^CD103^+^ DC1 dendritic cells, and F4/80^-^CD24^+^MHCII^+^Ly6C^-^CD11b^+^CD103^-^ DC2 dendritic cells as previously described (18), as well as CD4^+^ and CD8^+^ T cells (**Figure 2A**). Moc2 and SCC-VII tumors exhibited the lowest CD8^+^ and CD4^+^ T cell infiltrates, while SCC-VII demonstrated the highest infiltrate of TAM. Moc2 was characterized by the lowest overall immune cell infiltration, with the dominant infiltrating cell type being neutrophils in this tumor. Moc1, Moc1-ova, and TC1 exhibited the most favorable T cell infiltrate, with both CD8^+^ and CD4^+^ T cells present (**Figure 2B**). To better understand the regulatory pressure on the T cells, we used the IHC and flow cytometry data to examine a series of immune cell ratios that have been shown to impact the outcome in HNSCC – Neutrophil:T cell ratio; TAM : CD8 ratio; Monocyte : CD8 ratio, and FoxP3:CD8 ratio. Notably, SCC-VII demonstrated an extremely high TAM:CD8 ratio, which has been associated with a poor outcome in patients, while Moc1 and Moc1-ova exhibited little evidence of myeloid suppression (**Figures 2C, D**).

### Links Between the Cytokine and Chemokine Balance Produced in Tumor Explants and Immune Infiltration

To understand how these infiltrating cells impact the cytokine and chemokine environment of the tumor, and its response to immunotherapy, viable tumor fragments were tested in explant culture for their cytokine production. Of the 16 replicates, 4 replicates were left untreated as a baseline analysis, and 3 groups of 4 replicates were treated with anti-PD1 or anti-CTLA4 blocking antibodies, or anti-OX40 agonist antibodies. Following 24 hours of incubation at 37°C, the supernatants were collected and analyzed for secreted cytokines levels by multiplex bead assay.

**Figure 3:**
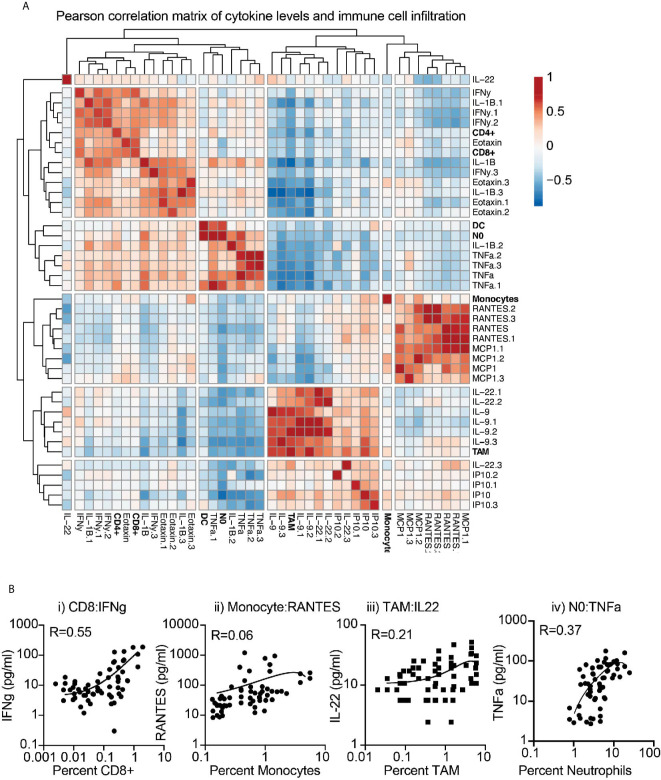
Correlation between immune infiltration and baseline cytokine and chemokine secretion from tumor explants. **(A)** Baseline cytokine and chemokine secretion detected by multiplex assay and matching immune infiltration determined by flow cytometry were analyzed for their correlation. Pearson correlation scores were clustered to identify potential coregulated features. Rows are centered; no scaling is applied to rows. Both rows and columns are clustered using maximum distance and average linkage. 42 rows, 42 columns. **(B)** Examples of correlated cytokines or chemokines with i) CD8 T cells, ii) Monocytes, iii) macrophages (TAM), and iv) Neutrophils (NO).

To first determine the association between baseline cytokine profiles and the tumor environment, we applied a correlation matrix between cytokine and chemokine levels and the infiltrating immune cells of the corresponding tumor, across multiple models. Correlations were further clustered to visualize associations (**Figure 3A**). Clear positively and negatively associated cells and cytokines were identified, with CD8 and CD4 T cell infiltrates positively correlated with IFNg and Eotaxin levels in the tumor, monocytes positively correlated with RANTES (CCL5) and MCP1 (CCL2) levels, and neutrophils positively correlated with IL1b and TNFa levels. These fit with established literature on the recruitment or cytokine production by these cells, but the positive correlation between TAM numbers and IL-9 and IL-22 levels was unexpected. To better understand these correlations, we plotted the cytokine/chemokine levels and infiltrating cell numbers for these key features, for each individual tumor across models (**Figure 3B**). The data demonstrates a strong relationship between CD8 and IFNg, but more variability between monocytes and RANTES, between TAM and IL22, and between neutrophils and TNFa across individual tumors. Together, these data indicate that explants can provide a snapshot of biological responses ongoing in tumors and provide information linked to the degree of immune infiltration and immune function in the tumor environment.

### Relationship Between Immuno-Infiltration and Cytokine/Chemokine Co-Secreted Networks Found in Tumor Explants Was Revealed by Surprisal Analysis

**Figure 4:**
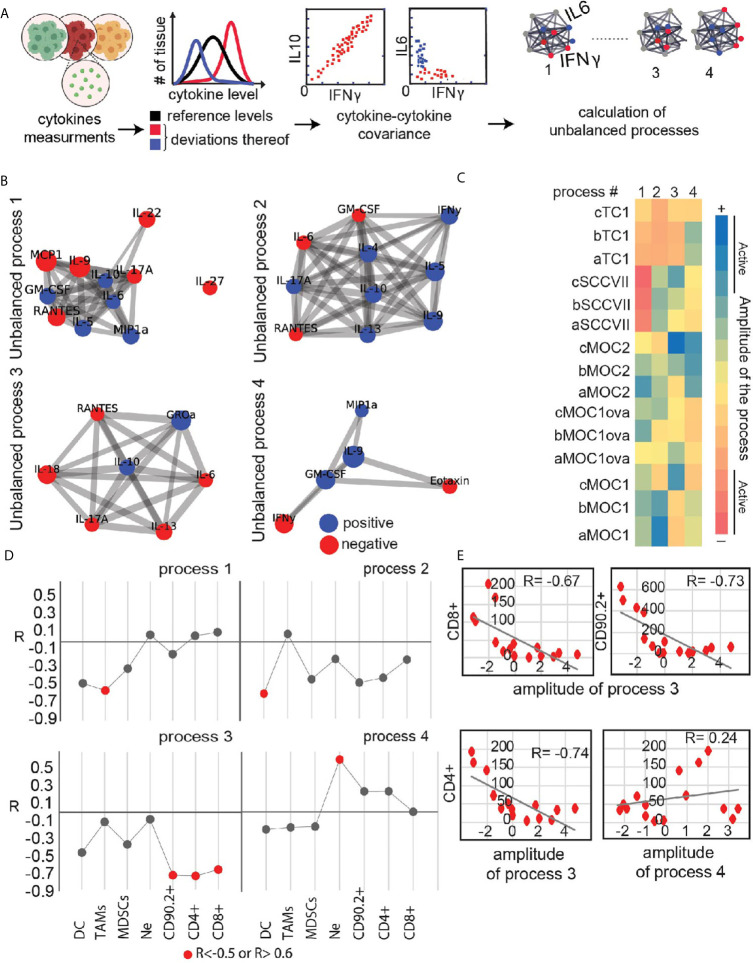
Utilizing secretome profiling to identify cytokine/chemokine expression patterns in different explants and their correlation with immune infiltrating cells. **(A)** Quantitative secretome data obtained from the cohort of HNSCC tumor models explants is used as an input for the analysis. Extent of variability in protein expression levels is quantified for each cytokine/chemokine. Protein distribution histograms are built to present and quantify this variability. Proteins whose expression levels deviate from the reference state in the same direction or in opposite directions, e.g. co-varying proteins, are grouped further into correlation networks (exemplified here by IFNg, IL10 and IL6). In this example IFNg and iL10 are correlated, whereas the expression levels of IFNg and IL6 are anticorrelated and deviate from the steady state in an opposite manner. Based on these co-varying groups of proteins, altered protein-protein correlation networks are generated (right panel). Several different protein-protein correlation networks can be found in the entire population of cancer explants. **(B)** Four significant unbalanced processes were found in the explant dataset based on error calculation. Cytokines with significant G_iα_ values (the cutoff was based on 20-fold difference between the 20% of cytokines with lowest G_iα_ in this process and the cytokines considered to be involved in the process) were assembled into networks using functional interactions according to STRING database. **(C)** Heatmap presents amplitude values of the 4 unbalanced processes for all tumor types. Threshold limits (indicated as “active” in the color chart) were calculated as described (22). **(D, E)** Correlation plots between amplitudes of the unbalanced process and the levels of biomarkers representing infiltrating immune-cells were generated for each process **(E)** shows examples of such plots showing a correlation between process 3 and CD4^+^, CD8^+^, and CD90.2^+^ infiltrating cells. **(D)** summarizes R correlation coefficient values between key immune infiltrating cells and unbalanced co-secretion processes.

To add an additional layer to the characterization of the immune state of different tumor explants we examined relationships between co-secretion patterns from the explants and infiltrating immune cells. First, we identified co-secretion altered subnetworks characterizing the explant dataset using surprisal analysis (21, 26). Input into the analysis are the expression levels of the secreted proteins measured in each tumor sample and then quantifies (**Figure 4A**) altered protein-protein subnetworks (unbalanced secretion processes) active in the entire dataset (**Figure 4B**). Four main unbalanced subnetworks were identified in the dataset of untreated tumor explants. The heatmap in **Figure 4C** presents the amplitude (importance) of each unbalanced process in each explant. For example, IL17, RANTES and IL22 are co-expressed and anti-correlated with IL6 and IL10 in unbalanced process 1 (**Figure 4B**). IL17, RANTES and IL22 are induced in SCCVII explants as indicated by the high amplitude calculated for process 1 in SCCVII explants (**Figure 4C**). The heatmap clearly distinguishes between TC1/SCCVII explants and Moc explants. However, there is also heterogenetity between individual tumors of a specific cell-line. For example, processes 2-4 are not active to a similar extent within the Moc1 explant subgroups, pointing to the basal heterogeneity in co-secretion patterns between the phenotypically similar tumors.

Next, we examined the relationship between the immune infiltrating cells and the four co-secretion patterns as represented by the unbalanced processes 1-4. We calculated an extension of correlation (R coefficient of correlation) between the biomarkers representing different immune cells and the amplitudes of the unbalanced processes. R value for each plot was calculated and all values were plotted in **Figure 4D** for major infiltrating populations. Induced cytokines due to process 3, labeled in red (such as RANTES and IL-6, **Figure 4C**) were correlated with high levels of CD4^+^, CD8^+^, and CD90.2^+^ cells (**Figures 4D, E**), whereas induced levels of GROa and IL-10 due to process 3 in Moc1 and Moc2 explants corresponded to the low levels of CD4^+^, CD8^+^, and CD90.2^+^ cells. As a comparison, a negative control representing a poor correlation between CD4^+^ and process 4 is shown (**Figure 4E**; lower and left panel). These data demonstrate that analysis of relationships between multiple markers present in explants can provide additional information and identify potential unbalanced processes that can be targeted to improve tumor immune environments.

### Response of Explants to Immunotherapy

**Figure 5:**
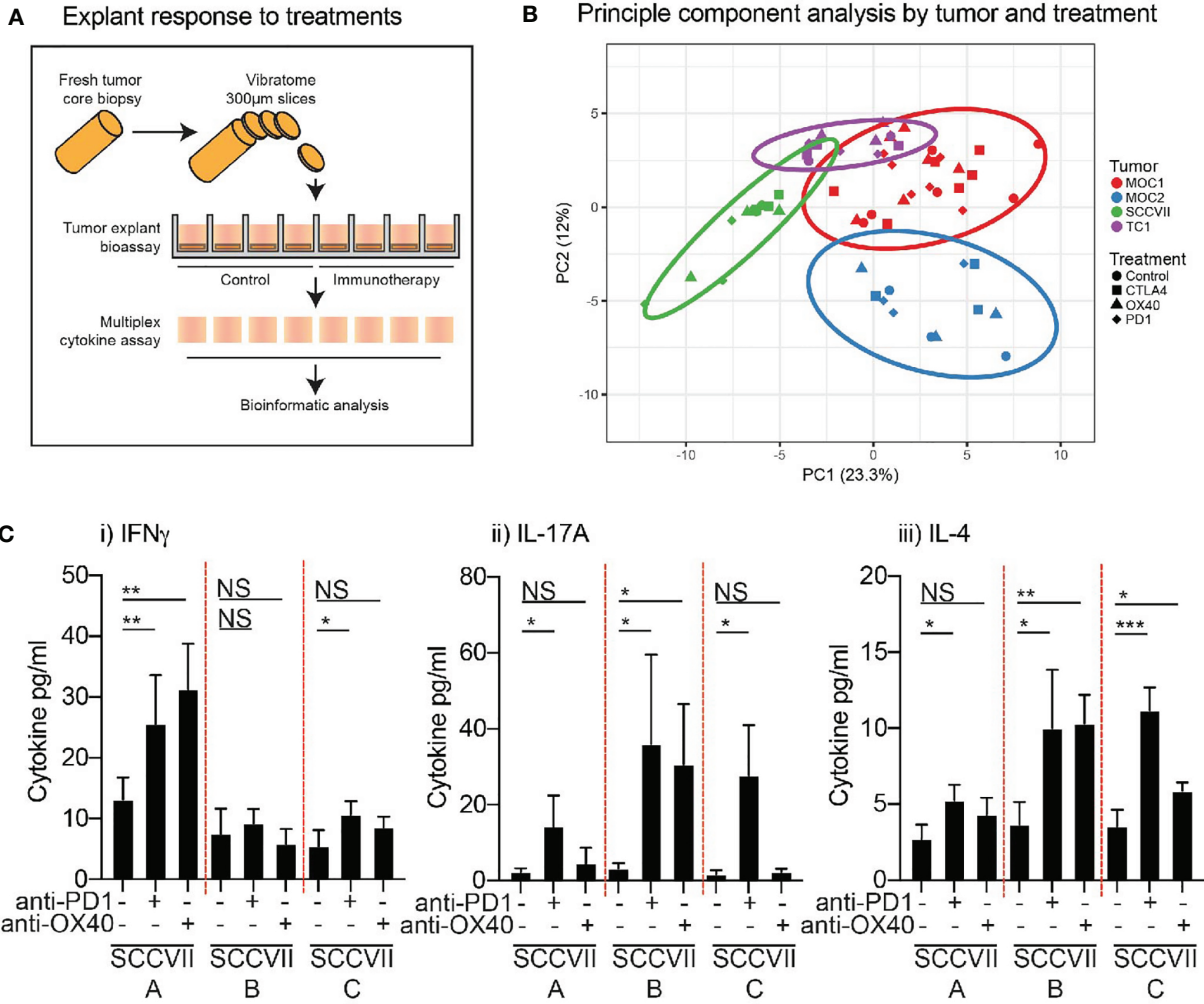
Effect of immunotherapy treatment on cytokine and chemokine secretion from tumor explants. **(A)** Outline of treatment method using intact tumor explants treated *ex-vivo* with candidate immunotherapies. **(B)** Cytokine and chemokine secretion detected by multiplex assay was evaluated by principal component analysis to identify potential distinguishing features. Colors show explants from a specific tumor type. Shapes show different treatments. **(C)** The response of individual SCC-VII tumor explants to treatment with anti-PD1 or anti-OX40 showing secretion of i) IFNg, ii) IL-17A, iii) IL-4. Graphs show mean and SD expression across replicate explants from individual tumors. Key: NS, not significant. *p<0.05; **p<0.01; ***p<0.001.

Next, to evaluate the response of tumor explants to immunotherapy, we assessed the effect of each agent on cytokine and chemokine production by the tumor explants (**Figure 5A**). To confirm the viability and activation status of lymphocytes in the tumor explants following treatment, tumors were harvested at 24 hours and individual tumor fragments were analyzed by flow cytometry (**Supplementary Figure 1A**). Despite the small size, we were able to identify critical immune cell subsets in the explants, and these retained the differential immune environments seen in the tumors at baseline, so Moc2 tumors exhibited lower overall lymphocyte infiltration and a lower CD8 T cell infiltration (**Supplementary Figure 1B**). To understand the complex cytokine and chemokine dataset, we attempted to identify treatment responses by clustering cytokine and chemokine levels across multiple treatments and multiple models. Principal component analysis was readily able to distinguish the individual tumor types being treated but the effect of the treatments was not detectable (**Figure 5B**). Similarly, cluster analysis readily clustered tumor types according to their unique cytokine and chemokine pattern, but the effect of treatment was not detectable (data not shown). Importantly, we were not able to detect differences in the surface activation markers tested by flow cytometry (**Supplementary Figure 1C**), suggesting that changes in cytokine and chemokine production may be a direct response to T cell stimulation rather than overall changes in T cell activation in the explants. Closer examination of the cytokine and chemokine data demonstrated that the limited ability to detect the effect of treatment on cytokine and chemokine production may be due to inter-individual variability in the response to therapy. The most consistent response was observed in SCC-VII tumors following treatment with anti-PD1 and anti-OX40 treatment, while these tumors showed limited effects following anti-CTLA4 treatment. When we examine the responses of individual tumors, we see that some tumors increase IFNg production following treatment, others increase IL-17 and IL-4 production (**Figure 5C**), despite very similar baseline levels of these cytokines. These data highlight significant inter-individual responses to identical therapies in identical tumor types. Since IFNg, IL17, and IL-4 production represent key markers of Th1, Th17, and Th2 subtypes, these responses may result from divergent differentiation of T cells in the individual tumors, each being derepressed or activated following anti-PD1 or anti-OX40. This is consistent with the ability of these agents to support the function of fully differentiated cells while remaining agnostic to their polarization (27–31). As shown, there is no evidence that these tumors were different at baseline, so the differing response to therapy was not predictable. Yet an IFNg-pattern of response is associated with improved outcome following anti-PD1 blockade in patients (32), suggesting that the explant response may help predict the response to patient treatment.

### Biological Response of HNSCC Patient Tumor Explants

**Figure 6:**
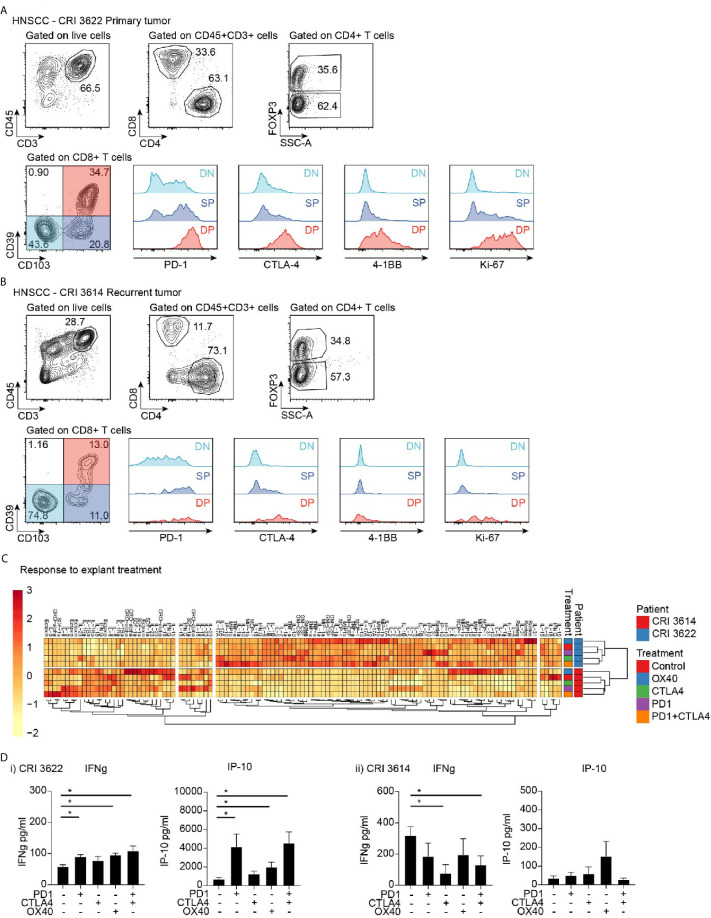
Effect of immunotherapy treatment on cytokine and chemokine secretion from tumor explants. Flow cytometry analysis of infiltrating T cell populations in HNSCC patients with **(A)** primary tumor and **(B)** recurrent tumor. Gating identifies CD4 and CD8 T cell infiltrate, as well as FoxP3^+^ Treg cells, and evaluates critical functional markers on the different CD8 T cell populations identified by CD39 and CD103 expression. **(C)** Cytokine and chemokine secretion from tumor explants from patients in **(A, B)** treated with candidate immunotherapies. Responses are clustered to identify potential coregulated differences. Rows are centered; unit variance scaling is applied to rows. Imputation is used for missing value estimation. Both rows and columns are clustered using correlation distance and average linkage. 120 rows, 8 columns. **(D)** Explant production of IFNg and IP10 in i) primary tumor and ii) recurrent tumor. Key: NS, not significant. *p < 0.05.

To determine whether the explant approach could be applied to patients, explants were prepared from resected human HNSCC from patients that underwent treatment in our institute. Each tumor was analyzed for baseline cytokine profile, and its replicate explants were also treated with various therapeutic agents. We are presenting here representative data from two patients, CRI3614 and CRI3622. CRI3614 was an advanced, recurrent tumor, while CRI3622 was a primary tumor. Analysis of the tumor microenvironment by flow cytometry revealed that the primary tumor showed a higher frequency of CD3^+^ and CD8^+^ cells, while the proportion of CD4^+^Foxp3^+^ cells was not different from the recurrent tumor (**Figures 6A, B**). Importantly, the primary tumor demonstrated a much higher proportion of CD103^+^CD39^+^ double-positive CD8^+^ T cells, a cell population associated with improved prognosis in HNSCC (7). Analysis of the explant responses showed a distinct baseline cytokine profile, suggesting that these patients have a distinct tumor environment (**Figure 6C**). As with the murine tumors, the patients are readily distinguished by cluster analysis using their cytokine and chemokine profile. While baseline IFNg is higher in the recurrent tumor, the level of TNFa, RANTES (CCL5), IP-10, IL-5, IL-10, MIP1a (CCL3), MIP1b (CCL4), and GM-CSF are all significantly lower in the recurrent tumor. These data suggest the existence of a more favorable immune environment in the primary tumor as compared to the recurrent tumor.

To understand the response to treatment in these patients, we analyzed the cytokine profile following treatment of explants with immunotherapeutic agents: PD-1 blockade, CTLA-4 blockade, a combination of PD-1 and CTLA-4 blockade, and OX40 stimulation. Notably, the patients differed in their responses. The primary tumor responded to PD1, OX40, and PD1+CTLA4 treatment with increased production of IFNg, and accompanying increases in the IFN-regulated chemokine IP-10 (CXCL10) (**Figure 6Di**) and the proinflammatory chemokine RANTES (not shown). By contrast, no increase in those cytokines/chemokines was observed in the recurrent tumor (**Figure 6Dii**), which was broadly unresponsive to treatment. Based on the IFN response pattern associated with success to PD-1 blockade (32), we would anticipate a more favorable response to treatment in the patient with the primary tumor. These data demonstrate that analysis of tumor explants has the potential to provide a rapid prediction of treatment responses in patients, and could be developed to select the most appropriate immunotherapy agent, or guide alternative options for unresponsive patients.

## Discussion

We present a method that utilizes fresh tumor tissue for conducting both baseline and response-to-treatment assays to bridge the gap between our understanding of the tumor environment and the response to treatment in the clinical setting. Our data from *in-vivo* HNSCC models shows that tumor environments differ in their immune infiltrate composition, amount, and spatial organization. Features such as neoantigen burden (33–36), T cell infiltration (37–40), and PDL1 expression (41–46) can be predictive of outcome in HNSCC and other cancers. The major issue is that in each of these examples there are individuals with low neoantigen burden, low T cell infiltration, and low PDL1 expression who respond to treatment, and conversely patients with all the positive features who can be unresponsive. There is a limited timeline in which alternative therapies could be evaluated in a patient who has failed first-line immunotherapy, and simultaneous combinations of therapies may limit their efficacy (47). We propose that analysis of the biological responses of fresh tumor explants will provide a novel way to potentially identify how a patient will respond to treatment, and through additional phenotyping of the tumor, identify key features of the tumor that dictate that response pattern.

To corroborate and complement our method, we used well-established experimental approaches – multiplex IHC, and flow cytometry. IHC depicts the spatial organization of immune cells infiltrating the tumor environment, and a growing amount of evidence underlines its potential as a tool to link the immune environment with clinical responses in HNSCC (9, 48) and other cancers (49). Flow cytometry permits a more comprehensive phenotypic characterization of the infiltrating immune cells while losing spatial information, and has been used to identify key prognostic cells in HNSCC (7). However, neither can provide functional information on the response to treatment, and our data demonstrate that the baseline immune status of the tumor is not necessarily predictive of the anti-tumor response.

The Surprisal analysis presented here allows us to find a relationship between co-secretion cytokine patterns and immune infiltrating cells. Although a high correlation between certain immune cells and co-secretion patterns was found (such as a correlation between CD4^+^, CD8^+^, and CD90^+^ cells and process 3, or a correlation between TAM cells and process 1) more research is required to establish those correlations further and investigate their mechanism. Another interesting finding points to a high basal heterogeneous environment of the examined tumors. Certain phenotypically similar tumors with the same origin (e.g. Moc1 subgroup) differ in the unbalanced processes they harbor (e.g. processes 2 and 3) as well as the number of immune-infiltrating cells, such as CD8^+^ and CD4^+^ cells. Analysis of large oral cancer datasets will be necessary to find features such as co-secretion patterns that may allow us to tailor personalized immunotherapies based on such explant analyses.

It remains necessary to correlate the explant response to treatment with *in-vivo* responses to the same treatment to validate this method in patients and animals. While the candidate immunotherapies tested here are effective in many murine models when given immediately following tumor implantation, they are ineffective as single agents in large, established tumors. Clinically, of the immunotherapies tested only PD1 blockade is approved for treatment in HNSCC. These issues limit our current ability to pair explant responses with clinical outcomes outside of clinical trials. In addition, our studies only explore cytokine and chemokine responses, which may be insufficient to fully model the biological response to treatment in the tumor. Cytokine and chemokine analysis may need to be combined with further analysis of immune cell activation or cancer cell death in the explant using alternative approaches. This may be particularly relevant for treatments such as anti-CTLA4, which has been proposed in preclinical models to function primarily as a Treg depleting therapy (50, 51). While we did not observe Treg depletion by anti-CTLA4 in the explants, additional timelines should be considered.

One strong caveat in the explant approach is that it is dependent on the response pattern of immune cells already present in the tumor environment. If the immunotherapy is dependent on the activation of immune cells in the circulation, or in secondary lymphatics, the explant approach may miss critical responses (52). As discussed above, many of the critical immune cells are already resident in tumors (7) and some therapies can function without systemic circulation of T cells (5, 6, 53). However, this is unlikely to be true for all cancer immunotherapies therefore the explant assay must be judiciously applied. For example, it is likely that vaccine-based immunotherapies require functional secondary lymphoid organs, and therefore such immunotherapies cannot be evaluated using explant tumors. However, since vaccine-based therapies still aim to change the tumor immune environment and can be used together with checkpoint therapies to overcome resistance at the cancer cell level or within the stromal environment (54–57), explant analysis may be applicable after vaccination to determine whether vaccine-based increase in circulating antigen-specific T cells has successfully resulted in a change in the biological responsiveness of the tumor immune environment.

At present, the explant assay is dependent on samples obtained at the time of surgical resection. Modifying the assay to use biopsy specimens could provide options for earlier prediction of response, and also provide opportunities for serial analysis of tumor responses to guide timing of serial immune interventions (47). In addition, with the increasing movement of immunotherapy towards the neoadjuvant setting in HNSCC (58) and other solid tumor types, biopsy-based prediction of response would permit rapid adaptive studies based on individual patient responses to available treatment options. We would advocate incorporating biological assays of response into ongoing clinical trials of novel immunotherapies to identify whether therapies can be better matched to responsive patients as the number of immunotherapy options continues to expand.

## Data Availability Statement

The raw data supporting the conclusions of this article will be made available by the authors, without undue reservation.

## Ethics Statement

The studies involving human participants were reviewed and approved by the Providence Portland Medical Center IRB (IRB# 12-075) for the provision of de-identified human tissue. The patients/participants provided their written informed consent to participate in this study. The animal study was reviewed and approved by Earle A. Chiles Research Institute IACUC (Animal Welfare Assurance No. A3913-01).

## Author Contributions

Conceptualization: MG and SS. Investigation: SS, TD, SB, JB, SV, and MJ. Methodology: SS, TD, MG, and NK-B. Resources: MG, MC, and BB. Visualization: MG and NK-B. Writing: SS, TD, RL, MC, NK-B, and MG. Funding acquisition: SS, MG, MC, NK-B. Supervision: MG and NK-B. All authors contributed to the article and approved the submitted version.

## Funding

This work was supported by NCI R01CA182311, NCI R01CA244142 (MG), NCI R01CA208644 (MC), Israel Cancer Association (SS), and Israel Science Foundation (NK-B).

## Conflict of Interest

MG and MC receive research funding from Bristol Myers-Squibb, Jounce, and Mavupharma that is unrelated to the content of this manuscript.

The remaining authors declare that the research was conducted in the absence of any commercial or financial relationships that could be construed as a potential conflict of interest.

## Supplementary Material

Supplementary Figure 1 Flow cytometry of tumor explants. **(A)** Treated and control explants were harvested after 24 hours and individually digested for flow cytometry. Flow cytometry gating to identify CD90+ lymphocytes, CD4+ and CD8+ T cells, and CD4+CD25+ cells. **(B)** Quantification of infiltrating cells in each explant. **(C)** Representative ICOS expression on T cell populations, and quantification of ICOS mean fluorescence intensity in T cells in explants.
