# Supplementary figures and images for: Explant Modeling of the Immune Environment of Head and Neck Cancer
Source: Front Oncol. 2021 Jun 17;11:611365. doi: 10.3389/fonc.2021.611365 (PMC8249923; doi:10.3389/fonc.2021.611365)

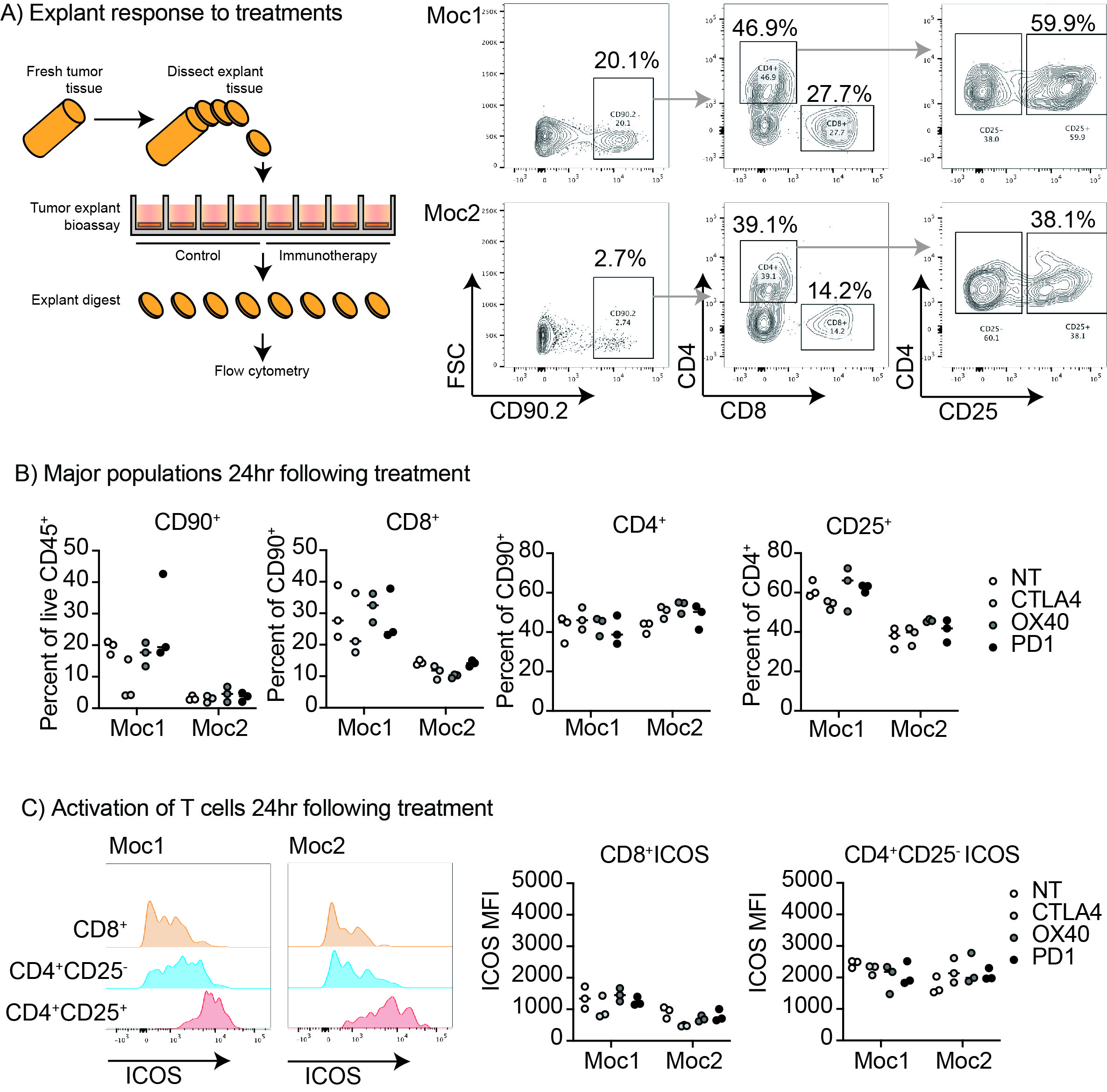

Supplement: Supplementary Figure 1 — Flow cytometry of tumor explants. (A) Treated and control explants were harvested after 24 hours and individually digested for flow cytometry. Flow cytometry gating to identify CD90+ lymphocytes, CD4+ and CD8+ T cells, and CD4+CD25+ cells. (B) Quantification of infiltrating cells in each explant. (C) Representative ICOS expression on T cell populations, and quantification of ICOS mean fluorescence intensity in T cells in explants. [file Image_1.jpeg]
